# The diabetic myocardial transcriptome reveals Erbb3 and Hspa2 as a novel biomarkers of incident heart failure

**DOI:** 10.1093/cvr/cvae181

**Published:** 2024-08-24

**Authors:** Marcella S Conning-Rowland, Marilena Giannoudi, Michael Drozd, Oliver I Brown, Nadira Y Yuldasheva, Chew W Cheng, Paul J Meakin, Sam Straw, John Gierula, Ramzi A Ajjan, Mark T Kearney, Eylem Levelt, Lee D Roberts, Kathryn J Griffin, Richard M Cubbon

**Affiliations:** LIGHT Laboratories, Leeds Institute of Cardiovascular and Metabolic Medicine, The University of Leeds, Leeds, UK; LIGHT Laboratories, Leeds Institute of Cardiovascular and Metabolic Medicine, The University of Leeds, Leeds, UK; LIGHT Laboratories, Leeds Institute of Cardiovascular and Metabolic Medicine, The University of Leeds, Leeds, UK; LIGHT Laboratories, Leeds Institute of Cardiovascular and Metabolic Medicine, The University of Leeds, Leeds, UK; LIGHT Laboratories, Leeds Institute of Cardiovascular and Metabolic Medicine, The University of Leeds, Leeds, UK; LIGHT Laboratories, Leeds Institute of Cardiovascular and Metabolic Medicine, The University of Leeds, Leeds, UK; LIGHT Laboratories, Leeds Institute of Cardiovascular and Metabolic Medicine, The University of Leeds, Leeds, UK; LIGHT Laboratories, Leeds Institute of Cardiovascular and Metabolic Medicine, The University of Leeds, Leeds, UK; LIGHT Laboratories, Leeds Institute of Cardiovascular and Metabolic Medicine, The University of Leeds, Leeds, UK; LIGHT Laboratories, Leeds Institute of Cardiovascular and Metabolic Medicine, The University of Leeds, Leeds, UK; LIGHT Laboratories, Leeds Institute of Cardiovascular and Metabolic Medicine, The University of Leeds, Leeds, UK; LIGHT Laboratories, Leeds Institute of Cardiovascular and Metabolic Medicine, The University of Leeds, Leeds, UK; LIGHT Laboratories, Leeds Institute of Cardiovascular and Metabolic Medicine, The University of Leeds, Leeds, UK; LIGHT Laboratories, Leeds Institute of Cardiovascular and Metabolic Medicine, The University of Leeds, Leeds, UK; LIGHT Laboratories, Leeds Institute of Cardiovascular and Metabolic Medicine, The University of Leeds, Leeds, UK

**Keywords:** Diabetes, RNA-seq, Erbb3, Hspa2, Heart failure, Myocardium

## Abstract

**Aims:**

Diabetes mellitus (DM) increases heart failure incidence and worsens prognosis, but its molecular basis is poorly defined in humans. We aimed to define the diabetic myocardial transcriptome and validate hits in their circulating protein form to define disease mechanisms and biomarkers.

**Methods and results:**

RNA-sequencing data from the Genotype-Tissue Expression (GTEx) project was used to define differentially expressed genes (DEGs) in right atrial (RA) and left ventricular (LV) myocardium from people with vs. without DM (type 1 or 2). DEGs were validated as plasma proteins in the UK Biobank cohort, searching for directionally concordant differential expression. Validated plasma proteins were characterized in UK Biobank participants, irrespective of diabetes status, using cardiac magnetic resonance imaging, incident heart failure, and cardiovascular mortality. We found 32 and 32 DEGs associated with DM in the RA and LV, respectively, with no overlap between these. Plasma proteomic data were available for 12, with ERBB3, NRXN3, and HSPA2 (all LV hits) exhibiting directional concordance. Irrespective of DM status, lower circulating ERBB3 and higher HSPA2 were associated with impaired LV contractility and higher LV mass. Participants in the lowest quartile of circulating ERBB3 or highest quartile of circulating HSPA2 had increased incident heart failure and cardiovascular death vs. all other quartiles.

**Conclusion:**

DM is characterized by lower Erbb3 and higher Hspa2 expression in the myocardium, with directionally concordant differences in their plasma protein concentration. These are associated with LV dysfunction, incident heart failure, and cardiovascular mortality.

## Graphical Abstract

**Graphical Abstract:**
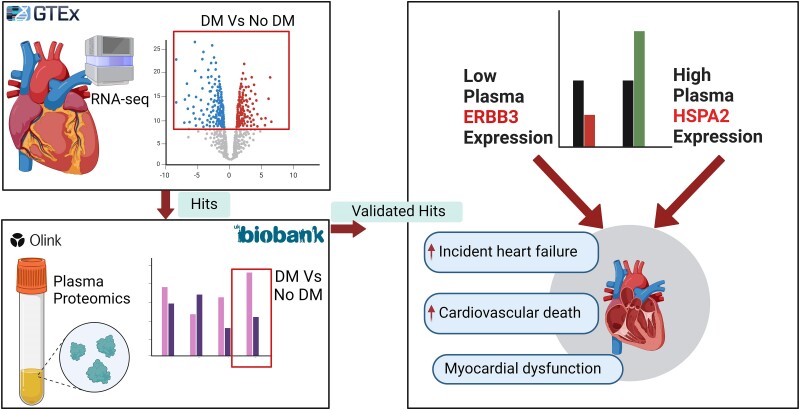
Created with BioRender.com.

**Time of primary review: 24 days**

**See the editorial comment for this article ‘Erbb3 and Hspa2, two novel predictors of heart failure in diabetic patients', by M. Forte *et al*., https://doi.org/10.1093/cvr/cvae220.**

## 1. Introduction

The global prevalence of diabetes mellitus (DM) was estimated at 9.3% of adults (463 million people) in 2019 and is expected to rise to 10.9% (700 million people) by 2045.^1^ While the incidence of most microvascular and macrovascular complications of DM continues to decline, the incidence of heart failure (HF) is static.^2^ Epidemiological studies suggest that people with DM experience at least a doubled incidence of HF, both for those with type 1 or type 2 DM, with comparable incidence rates for HF with preserved or reduced ejection fraction.^3,4^ Moreover, the prognosis of people with HF who also have DM is worse, with doubled rates of all-cause mortality^5^ and 50% greater rates of HF hospitalization, compared to individuals with normal glucose metabolism.^6^ While obesity is an important risk factor for HF in people with DM, it is notable that even those people without obesity or other modifiable risk factors experience increased risk of HF and myocardial dysfunction.^2,7^ This suggests that existing risk factor modification strategies are not fully addressing the pathophysiology of diabetic heart disease.

Our understanding of the molecular basis of myocardial disease associated with DM largely comes from animal models that do not recapitulate many of the phenotypic and treatment characteristics of people with DM.^8–10^ This reflects the challenges of collecting myocardial biopsies, especially of the left ventricle, from large cohorts of people with and without DM. Moreover, many studies apply a biased approach to molecular characterization, focusing on predefined genes or pathways, which further increases the risk of missing unappreciated pathophysiological processes. We aimed to address this by using RNA-sequencing (RNA-seq) data from the Genotype-Tissue Expression (GTEx) project, which collected multi-organ biopsy material from a large cohort of organ donors.^11^ Differentially expressed genes (DEGs) associated with DM were defined separately in right atrial (RA) and left ventricular (LV) tissue. These were further explored using plasma proteomic data from the UK Biobank (UKB) cohort to seek concordant hits with potential use as biomarkers and to inform understanding of pathophysiology.

## 2. Methods

### 2.1 Myocardial RNA-seq data from GTEx project

The GTEx project was established by the Broad Institute of MIT and Harvard with a primary goal of defining associations between genomic variation and gene expression in 54 tissues across the human body.^11^ Tissues were retrieved post-mortem from organ donors following consent from family decision-makers. RNA-seq was performed as described in detail on the GTEx project website (https://gtexportal.org/home/methods). RA appendage and LV apex RNA-seq data are available from 429 and 432 donors, although our analyses pertain to 425 RA and 428 LV samples after exclusion due to missing metadata required as covariates in differential gene expression analysis. Bulk RNA-seq raw count data were downloaded directly from the GTEx portal (https://www.gtexportal.org/home/), using GTEx version 8 release on 18 July 2022. This includes metadata on donor age, sex, ischaemic time (between death and sample collection), and Hardy score (mode of death classification). For sensitive metadata, pertaining to comorbidities, a protected access data application was granted by dbGaP (https://www.ncbi.nlm.nih.gov/gap/) #32524 ‘Defining tissue-specific transcriptional profiles associated with diabetes’.

### 2.2 RNA-seq analysis pipeline for GTEx data

Differential gene expression analysis was conducted using R (Version 4.1.1) and the DESeq2 package (Version 1.36.0).^12^ We sought DEGs associated with diabetes. Donors with type 1 or type 2 diabetes were pooled to create a single diabetes variable, since multiple donors were recorded as having both forms of diabetes and detailed treatment data were not available. Those with diabetes status unknown were excluded. Covariates in DESeq2 analyses were age (20–29, 30–39, 40–49, 50–59, 60–69, and 70–79); sex; race; ischaemic time as categories of 300 min (0–299, 300–599, 600–899, 900–1199, and 1200–1499); Hardy score; BMI which was classed as normal weight (18.5–24.9 kg/m^2^), overweight (25–29.9 kg/m^2^), and obese (>30 kg/m^2^); medical history indication of hypertension and myocardial infarction; and RNA integrity score (RIN; 5.1–6, 6.1–7, 7.1–8, 8.1–9, and 9.1–10). Technical factors including ischaemic time, defined by GTEx as ‘time from death or withdrawal of life-support until the time the sample is placed in a fixative solution or frozen’, Hardy score, and RIN were included. Biological cofactors of age, sex, race, and BMI were included because these are also known to alter cardiac gene expression. Genes were filtered to include only those with greater than 10 read counts in at least the same number of samples as included in the diabetes subgroup. Effect size shrinkage using the *apeglm* method was applied for visualization and ranking of genes.^13^ False discovery rate (FDR)–adjusted *P* values produced by DESeq2 using the Benjamini–Hochberg method were used, with adjusted *P* < 0.05 defined as statistically significant. Functional profiling of DEGs was performed using gProfiler (https://biit.cs.ut.ee/gprofiler/gost). DEGs were run as queries, with significance threshold calculated using Benjamini–Hochberg FDR. Driver terms were highlighted in the output as the most relevant Gene Ontology (GO) terms and exported.

### 2.3 UK Biobank cohort

UKB is a prospective observational cohort study of 502 462 participants aged 37–73 years, recruited from 22 assessment centres across the UK between 2006 and 2010. It is an open access resource developed using UK Government and biomedical research charity funding which linked wide-ranging phenotypic and healthcare record data. The UKB resource is open to all bona fide researchers. Full details of its design and conduct are available online (https://www.ukbiobank.ac.uk). UKB received ethical approval from the NHS Research Ethics Service (11/NW/0382); we conducted this analysis under application number 105351. All participants provided written informed consent, and the research was conducted in line with the Declaration of Helsinki. All analyses were conducted via the UKB Research Analysis Platform (https://ukbiobank.dnanexus.com).

### 2.4 Definition of diabetes in UKB

Baseline sociodemographic characteristics and comorbidities were recorded by participants completing a touchscreen and nurse-led interview at study recruitment and used as we have previously described.^14^ Data from face-to-face nurse-led interview was used to ascertain baseline comorbidities and medication. Diabetes was classified as any of ‘diabetes’ (UK Biobank field ID ‘1220’); ‘type 1 diabetes mellitus’ (‘1222’); ‘type 2 diabetes mellitus’ (‘1223’); ‘diabetic eye disease’ (‘1276’); ‘diabetic neuropathy/ulcers’ (‘1468’), and ‘diabetic nephropathy’ (‘1607’), as we have previously described.^7^

### 2.5 Plasma proteomic data in UKB

A recent update to UKB is the addition of plasma proteomic profiles from 54 219 UKB participants.^15^ These were measured with the Olink Explore 3072 panel, a proximity extension assay using paired antibodies and complimentary oligonucleotides, which quantifies 2923 unique proteins including clinically relevant biomarkers of cardiac stress and/or injury including NT-proBNP and TNNI3 (troponin I). Details of UKB quality control procedures for plasma proteomics have been descried by Sun and colleagues.^15^ Protein concentration is provided as normalized protein expression (NPX) values. Where proteins were below the limit of assay detection in specific samples, these are recorded as missing data. NPX values were converted to *Z*-scores, defined as *z* = (*x*−μ)/σ, where *x* = protein NPX, μ = population mean, and σ = population standard deviation. We used proteomic data collected at the initial assessment centre visit.

### 2.6 Cardiovascular magnetic resonance imaging data in UKB

From 2014, ∼50 000 participants have taken part in multimodality imaging assessment, which included cardiovascular magnetic resonance imaging (MRI).^16–18^ Expression of ERBB3 was compared against MRI parameters to determine if protein expression related to MRI markers of cardiac function. MRI parameters used were LV ejection fraction (LVEF; UKB field ID: ‘22 420’), LV circumferential strain global (UKB field ID: ‘24 157’), LV longitudinal strain global (‘24181’), LV radial strain global (‘24174’), LV myocardial mass (‘24105’), and myocardial wall thickness global (‘24140’). Of the UKB participants who had ERBB3 protein expression data, there were LV imaging data on ∼5000 of these (ranging from 4986 participants for LV longitudinal strain global to 5122 participants for LV myocardial mass). Additionally, cardiac contractility index (CCI) was used as an additional measure of cardiac functionality. CCI was derived by systolic blood pressure (UKB field ‘4080’) divided by LV end-systolic volume (LVESV) index, calculated as LVESV (‘22422’) normalized to body surface area (‘22427’) as previously described.^7^ When calculating CCI, six participants with LVESV < 20 mL were excluded as outliers of >3 standard deviations from the mean.

### 2.7 Definition of outcome measures in UKB

Time-to-HF was defined as the time between date of baseline assessment centre visit (when plasma proteins were measured) and the date that HF first reported (UKB field IDs 131354 and 131355), excluding participants with pre-existing heart failure. Self-reported HF codes were excluded (UKB code IDs 50 and 51 in data field 131355) to focus solely on events confirmed in medical records, including the death register, primary care, and hospital admission reports (UKB code IDs 20, 21, 30, 31, 40, and 41 in data field 131355). Cardiovascular mortality was defined I00–I99, excluding those related to infection mortality, as previously described.^7^

### 2.8 Statistical analysis of UKB

Categorical data are presented as number (percentage of denominator), and continuous data are presented as mean (standard deviation). Statistical significance was defined as *P* < 0.05 using two-sided tests. Normality was determined by skewness and kurtosis tests using the Moments (v0.14.1) R package (https://cran.r-project.org/web/packages/moments/index.html). Correlations between ERBB3 or HSPA2 and other continuous variables were calculated using Spearman's rank test. Comparisons across ERBB3 or HSPA2 quartile groups were made using *χ*^2^ tests followed by pairwise proportional tests for categorical data, and one-way ANOVA followed by Tukey *post hoc* tests for continuous data. Where a one-way ANOVA was not appropriate due to non-normality of data, Welch's ANOVA followed by Games–Howell *post hoc* tests were used as an alternative. Time-to-event analyses were performed using the R package Survival (v3.2.13),^19^ The censorship date for these analyses was 30 Aug 2023. As the proportional hazard assumptions were not met by Cox regression models, Poisson regression models including exposure time were generated instead. ROC curves were generated using the pROC package (1.18.5) using binary logistic regression models.^20^

## 3. Results

Bulk RNA-seq data from GTEx was used to identify differentially expressed genes (DEGs) in samples from people with DM vs. people without DM. Of RA donors 23.5% had DM, whilst this was 23.3% for LV donors. Overall, there was a greater proportion of men than women, and the majority of donors were white. There was a greater prevalence of prior myocardial infarction and hypertension in the DM donors (see Supplementary material online, *Table S1*). BMI, ischaemic time, and Hardy score were comparable in donors with and without DM. As all covariates in Supplementary material online, *Table S1*, have important implications for gene expression in individual samples, they were included as covariates in differential gene expression analyses, even if data were similar in groups with and without DM.

### 3.1 DEGs associated with diabetes in the LV and RA

**Figure 1:**
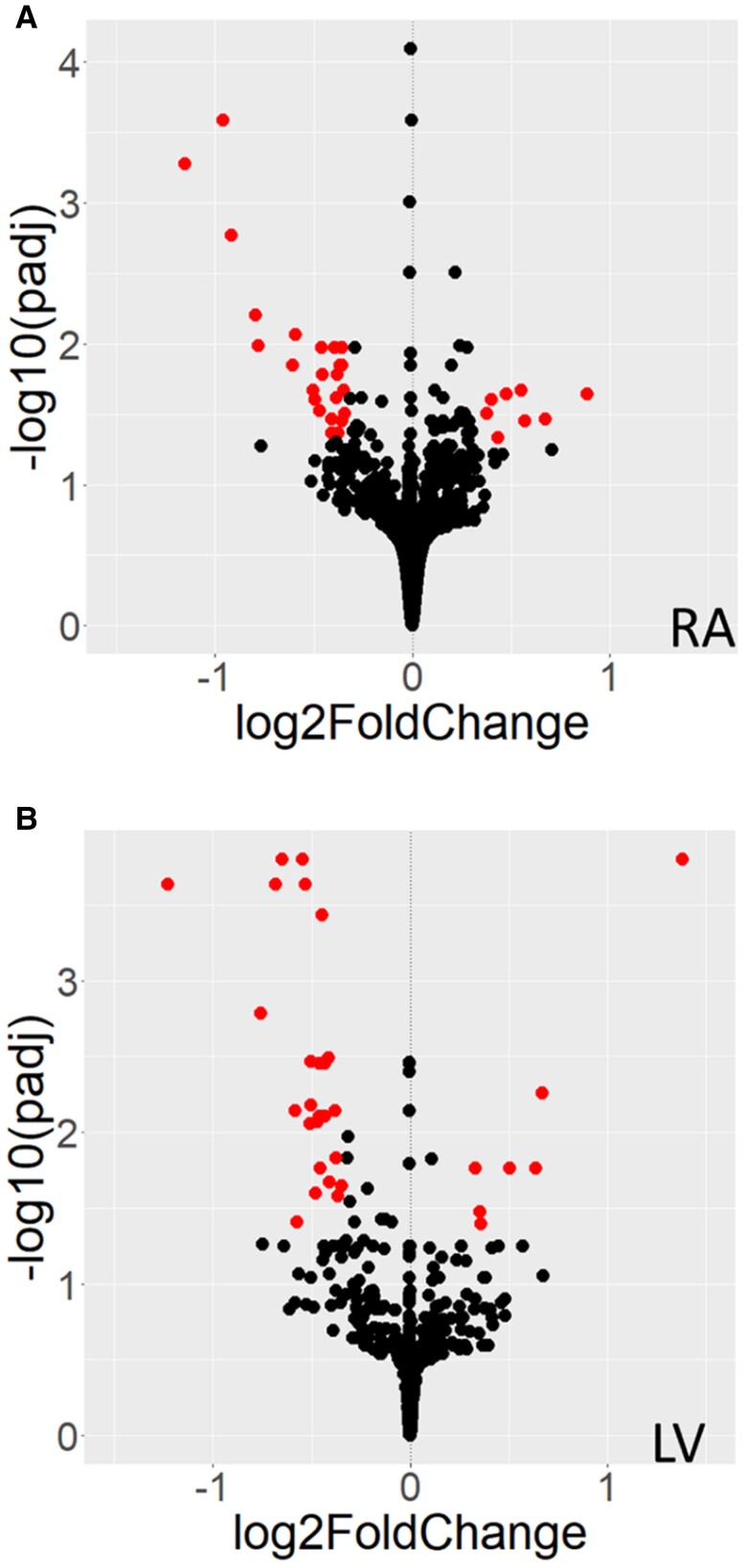
Differential gene expression associated with diabetes in the RA and LV. Volcano plots illustrating differential gene expression for DM vs. without DM for RA (*A*: *n* = 120 vs. 305 participants) and LV (*B*: *n* = 100 vs. 329 participants). Each dot represents a gene, with red colour denoting those that achieve Benjamini–Hochberg FDR-adjusted *P* < 0.05 and log_2_ fold change > 0.32 or < −0.32 (corresponding to a 25% difference in gene expression). Raw data are presented in Supplementary material online, *Tables S1* and *S2*. LV, left ventricle; RA, right atrium.

After accounting for confounding covariates, we identified 46 and 72 DEGs associated with DM (FDR-adjusted *P* < 0.05) in the LV and RA, respectively (*Figure 1*; Supplementary material online, *Tables S2*–S3). Of these, 32 LV and 32 RA DEGs had a log_2_ fold change > 0.32 or < −0.32 (corresponding to a 25% difference in gene expression, although our use of effect size shrinkage means differences are likely to be larger). Notably, there was no overlap in DEGs in the LV and RA, suggesting that DM has different pathophysiological implications in ventricular vs. atrial myocardium. To explore the biological themes within DEGs, functional enrichment analysis was performed using g:Profiler.^21^ In RA, this detected enrichment of immune system terms including IgG and IgA immunoglobulin complexes (GO terms: GO:0019814, GO:0071735, and GO:0071745). In LV, IgG immunoglobulin complex (GO:0071735) was also altered, but the several altered LV GO terms related to neuronal activity such as neuroligin family binding protein and structural constituent of myelin sheath (GO:0097109 and GO:0019911; see Supplementary material online, *Tables S4*–S5).

### 3.2 Validation of DEGs using UKB plasma proteomics

To corroborate the DEGs observed in GTEx, we used the orthogonal approach of defining these genes at their protein level in the circulation, which may also aid the translation of these findings to clinically relevant biomarkers. UKB used Olink technology to measure 2923 proteins in the plasma of 52 705 participants. Of the 64 DEGs observed in either LV or RA, proteomic data were available for 12 (*Table 1*). Eleven exhibited statistically significant differences in plasma protein expression between DM and non-DM participants. However, only ERBB3 (lower in DM), NRXN3 (lower in DM), and HSPA2 (higher in DM) exhibited a directionally concordant change in myocardial RNA expression and plasma protein concentration (*Table 1*). Moreover, DM was not associated with altered expression of these genes in non-cardiac tissues (see Supplementary material online, *Table S6*). Hence, these plasma proteins warrant further exploration as biomarkers of cardiac disease associated with DM.

**Table 1:** Validation of GTEx myocardial RNA-seq hits using UKB plasma proteomics

Gene	GTEx			UKB		
				No DM	DM	
	Tissue	pAdj	Log2 FC	*Z*-score	*Z*-score	pAdj
CGA	RA	0.028	0.89	0.006 (0.004)	−1.04 (0.004)	7.7e−11
MZB1	RA	0.010	−0.78	−0.02 (0.004)	0.352 (0.005)	3.2e−65
SLAMF7	RA	0.009	−0.59	−0.019 (0.004)	0.335 (0.005)	3.2e−65
COL28A1	LV	0.003	−0.5	−0.007 (0.004)	0.11 (0.005)	1.5e−7
PCDH7	RA	0.030	−0.46	−0.007 (0.005)	0.132 (0.005)	9.6e−10
ERBB3	LV	0.0004	−0.45	0.004 (0.004)	−0.068 (0.005)	8.4e−4
NRXN3	LV	0.004	−0.43	0.003 (0.005)	−0.052 (0.005)	9.8e−3
L1CAM	LV	0.003	−0.41	−0.009 (0.004)	0.162 (0.005)	1.0e−14
SLITRK2	LV	0.021	−0.41	−0.015 (0.004)	0.255 (0.005)	3.0e−38
WFDC1	RA	0.043	−0.38	−0.008 (0.005)	0.138 (0.005)	8.7e−13
HSPA2	LV	0.034	0.35	−0.014 (0.005)	0.238 (0.005)	3.2e−27
NUDT10	RA	0.031	−0.34	0.002 (0.005)	−0.029 (0.004)	NS

Z-scores presented as mean (SEM). GTEx analysis of RA represents 120 participants with DM vs. 305 participants without DM, and LA represents 100 participants with DM vs. 329 participants without DM. UKB analysis represents data from cohort of 52 705 participants.

DM, diabetes mellitus; FC, fold change; GTEx, Genotype-Tissue Expression project; NS, non-significant; UKB, United Kingdom Biobank.

### 3.3 Cardiovascular outcomes of directionally concordant hits in UKB

**Figure 2:**
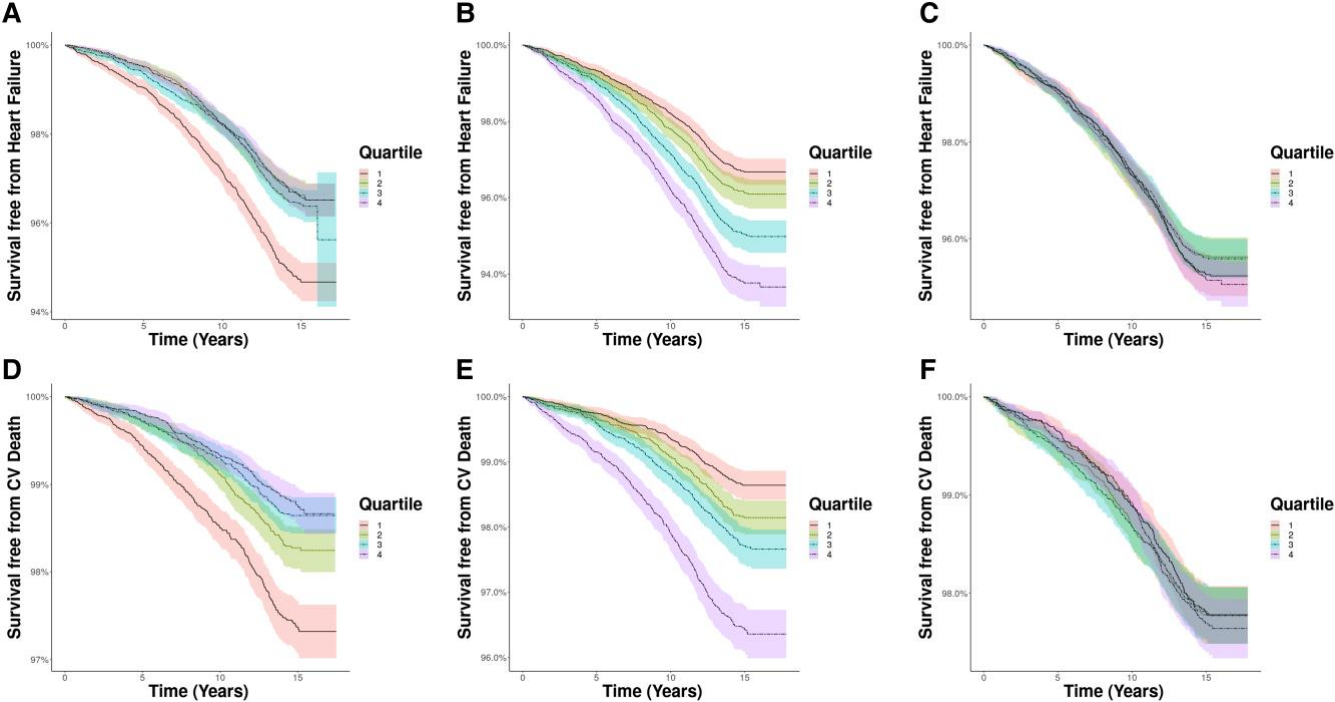
Incident HF and cardiovascular mortality in plasma ERBB3, HSPA2, and NRXN3 quartiles. Kaplan–Meier curves illustrating probability of freedom from HF (*A–C*) or cardiovascular mortality (*D–F*) during long-term follow-up of the UKB cohort stratified into quartiles of plasma ERBB3 (*A* and *D*), HSPA2 (*B* and *E*), and NRXN3 (*C* and *F*). Quartile 1 represents the lowest quartile of expression and quartile 4 the greatest; each quartile includes 12 833 participants for ERBB3, 11 114 participants for HSPA2, and 10 890 participants or NRXN3.

Next, we asked if plasma ERBB3, NRXN3, and HSPA2 were associated with long-term development of major cardiovascular events (2234 incident HF events during 719 262 person-years follow-up and 1139 cardiovascular deaths during 733 833 person-years follow-up). Kaplan–Meier curves illustrate a higher incidence of heart failure in the lowest quartile of ERRB3 (*Figure 2A*) and highest quartile of HSPA2 (*Figure 2B*); there were no interquartile differences for NRXN3 (*Figure 2C*). Kaplan–Meier curves again illustrate a higher incidence of cardiovascular mortality in the lowest quartile of ERRB3 (*Figure 2D*) and highest quartile of HSPA2 (*Figure 2E*); there were no interquartile differences for NRXN3 (*Figure 2F*). Hence, we prioritized our ongoing focus on ERBB3 and HSPA2.

Poisson regression was used to explore whether ERBB3 and HSPA2 remained associated with these events after accounting for potential confounding factors (*Table 2*). After adjustment for age, sex, BMI, SBP, and DM, adverse outcomes persisted in the lowest quartile of ERBB3 and highest quartile of HSPA2. However, further inclusion of NT-proBNP in these models resulted in loss of associations, except for higher cardiovascular mortality in the highest quartile of HSPA2. We also explored whether ERBB3 or HSPA2 added prognostic value to NT-proBNP as continuous variables using ROC analyses; these showed no statistically significant increase in the c-statistic, implying no added value to the already robust performance of NT-proBNP (see Supplementary material online, *Figures S1*–S2). However, in clinical practice, NT-proBNP is dichotomized to define high-risk populations, and so we asked if ERBB3 and HSPA2 were associated with adverse outcomes after excluding participants in the upper decile of NT-proBNP. This showed that cardiovascular mortality was higher in lowest quartile of ERBB3 and the highest quartile of HSPA2, after adjustment for age, sex, BMI, and diabetes (see Supplementary material online, *Table S7*), although there was no association with incident heart failure.

**Table 2:** Cardiovascular outcomes associated with plasma ERBB3 and HSPA2

Model	ERBB3 (Q1 vs. Q2–4)				HSPA2 (Q4 vs. Q1–3)			
	Incident heart failure		Cardiovascular mortality		Incident heart failure		Cardiovascular mortality	
	IRR (CI)	*P* value	IRR (CI)	*P* value	IRR (CI)	*P* VALUE	IRR (CI)	*P* value
Unadjusted	1.66 (1.53–1.81)	<2e−16	1.92 (1.71–2.17)	<2e−16	1.55 (1.4–1.71)	<2e−16	2.01 (1.76–2.29)	<2e−16
+sex	1.44 (1.32–1.58)	9.6e−16	1.53 (1.35–1.73)	2.1e−11	1.41 (1.28–1.56)	6.9e−12	1.76 (1.55–2.01)	<2e−16
+age	1.19 (1.08–1.3)	2.5e−4	1.22 (1.07–1.39)	2.5e−3	1.27 (1.15–1.4)	2.0e−6	1.59 (1.39–1.81)	6.2e−12
+BMI	1.2 (1.09–1.31)	1.6e−4	1.24 (1.09–1.41)	1.2e−3	1.17 (1.06–1.29)	2.0e−3	1.48 (1.29–1.69)	9.5e−9
+SBP	1.22 (1.1–1.34)	9.3e−5	1.34 (1.17–1.54)	2.9e−5	1.16 (1.05–1.28)	4.4e−3	1.45 (1.27–1.66)	5.0e−8
+DM	1.22 (1.11–1.34)	7.4e−5	1.23 (1.17–1.54)	2.4e−5	1.16 (1.05–1.29)	4.9e−3	1.5 (1.3–1.73)	1.9e−8
+NT-proBNP	1.02 (0.92–1.12)	NS	1.08 (0.94–1.24)	NS	1.08 (0.98–1.2)	NS	1.42 (1.23–1.64)	2.0e-6

Models represent incremental complexity by addition of each variable to all those listed above them. Each quartile includes 12 833 participants for ERBB3 and 11 114 participants for HSPA2.

IRR, incidence rate ratio; CI, confidence interval; BMI, body mass index; DM, diabetes mellitus; NS, non-significant; SBP, systolic blood pressure.

As ERBB3 signals by heterodimerizing with other ERBB family members, we conducted similar analyses for ERBB2 and ERBB4 as UKB proteomic data are also available for these. We found similar, although less robust, patterns for both ERBB2 and ERBB4, with participants in the lowest quartile having higher risks of incident heart failure and cardiovascular mortality than some other quartiles when adjusted for age, sex, BMI, and DM. (see Supplementary material online, *Figures S3*–S4 and *Tables S8*–S9).

### 3.4 Participant characteristics associated with ERBB3 and HSPA2 in UKB

To examine the wider characteristics of participants according to circulating ERBB3 or HSPA2 concentrations, we divided UKB participants into quartiles, with quartile 1 (Q1) representing the lowest concentration. As shown in *Table 3*, lower ERBB3 was associated with older age, male sex, marginally lower BMI, higher prevalence of DM, prior MI and HF, and greater use of a range of cardiovascular and diabetes medications. As shown in *Table 3*, higher HSPA2 was associated with older age, female sex, higher BMI, higher prevalence of DM, prior MI and HF, and greater use of a range of cardiovascular and diabetes medications.

**Table 3:** Baseline characteristics of plasma ERBB3 and HSPA2 quartiles

	ERBB3					HSPA2				
	Q1 (Low)	Q2	Q3	Q4 (High)	*P* value	Q1 (Low)	Q2	Q3	Q4 (High)	*P* value
Age (years)	57.8 (0.08)	56.5 (0.07)	56.4 (0.07)	56.7 (0.07)	<0.0001	55.7 (0.08)	56.5 (0.08)	57.2 (0.08)	57.9 (0.08)	<0.0001
Male (%)	67.2 (8618)	49.3 (6329)	38.2 (4912)	29.2 (3756)	<0.0001	65.3 (7274)	57.2 (6374)	51.6 (5746)	42.1 (4691)	<0.0001
BMI (kg/m^2^)	27.3 (0.04)	27.3 (0.04)	27.5 (0.04)	27.9 (0.04)	0.002	26.9 (0.04)	27.3 (0.04)	27.5 (0.05)	28.1 (0.05)	<0.0001
DM (%)	6.8 (869)	5.0 (643)	4.5 (579)	5.6 (724)	<0.0001	3.9 (439)	4.8 (535)	5.3 (583)	7.6 (847)	<0.0001
Prior MI (%)	4.6 (591)	2.4 (316)	1.9 (243)	1.7 (213)	<0.0001	1.8 (204)	2.4 (263)	2.6 (290)	3.6 (397)	<0.0001
Prior HF (%)	0.9 (102)	0.5 (69)	0.3 (45)	0.4 (47)	<0.0001	0.4 (46)	0.7 (82)	0.9 (100)	1.6 (179)	<0.0001
Systolic BP (mmHg)	136.4 (0.2)	136.8 (0.2)	137.9 (0.2)	139.8 (0.2)	<0.0001	136.0 (0.2)	137.0 (0.2)	138.0 (0.2)	140.0 (0.2)	<0.0001
Diastolic BP (mmHg)	80.6 (0.1)	81.6 (0.1)	82.5 (0.1)	83.7 (0.1)	0.002	81.2 (0.1)	82.1 (0.1)	82.5 (0.1)	82.9 (0.1)	<0.0001
ACE inhibitor (%)	11.5 (1473)	8.7 (1112)	7.9 (1016)	8.4 (1080)	<0.0001	7.2 (799)	7.8 (871)	9.2 (1022)	11.6 (1287)	<0.0001
Beta-blocker (%)	10.3 (1317)	6.9 (890)	6.0 (769)	6.3 (804)	<0.0001	6.1 (680)	6.1 (680)	7.7 (852)	9.1 (1015)	<0.0001
CCB (%)	8.7 (1114)	7.7 (985)	7.0 (893)	8.4 (1080)	<0.0001	6.2 (688)	6.9 (765)	7.8 (864)	10.5 (1168)	<0.0001
Thiazide (%)	6.7 (863)	6.8 (878)	7.2 (924)	8.8 (1134)	<0.0001	6.5 (724)	6.7 (747)	7.3 (818)	9.2 (1025)	<0.0001
Loop diuretic (%)	1.9 (244)	1.4 (185)	1.3 (167)	1.6 (206)	0.006	0.9 (104)	1.1 (120)	1.4 (151)	2.5 (283)	<0.0001
Metformin (%)	3.8 (491)	2.8 (357)	2.8 (365)	3.4 (436)	<0.0001	2.3 (261)	2.8 (308)	3.1 (347)	4.5 (502)	<0.0001
Sulphonylurea (%)	1.6 (203)	1.1 (147)	1.2 (151)	1.4 (174)	0.008	1.0 (109)	1.1 (119)	1.2 (131)	1.9 (208)	<0.0001
Insulin (%)	1.4 (178)	1.2 (156)	1.0 (123)	1.2 (151)	0.017	0.6 (64)	1.0 (108)	1.0 (108)	2.0 (217)	<0.0001

Data presented as mean (SEM) for continuous data and % (*n*) for categorical data, compared with ANOVA or *χ*^2^ tests, respectively. ERBBB3 quartiles were defined as Z-scores of below −0.628 for Q1, −0.628 to −0.013 for Q2, −0.013 to 0.595 for Q3, and above 0.595 for Q4; each quartile includes 12 833 participants. HSPA2 quartiles were defined as *Z*-scores of below −0.601 for Q1, −0.601 to −0.055 for Q2, −0.055 to 0.496 for Q3, and above 0.497 for Q4.

ACE, angiotensin-converting enzyme; BP, blood pressure; CCB, calcium channel blocker.

To assess association with baseline cardiac function, we defined correlation with cardiac MRI metrics (*Table 4*), focusing on measures of LV function given ERBB3 and HSPA2 were differentially expressed in LV myocardium. Lower ERBB3 was associated with markers of impaired LV contractility and higher LV mass; these correlations were weak, but of a magnitude similar to those noted for NT-proBNP (see Supplementary material online, Table 1*0*). These associations lost statistical significance when adjusting for age, sex, and indicators of current cardiometabolic disease. Similarly, higher HSPA2 was associated with impaired LV contractility and higher LV mass, albeit with weak correlation coefficients, and the association with LV mass persisted in adjusted analyses.

**Table 4:** Cardiometabolic biomarkers associations with ERBB3 and HSPA2 in UKB

	ERBB3			HSPA2		
	Unadjusted *R*	Unadjusted *P* value	Adjusted *P* value	Unadjusted R	Unadjusted *P* value	Adjusted *P* value
LVEF (%)	0.05	9.6e−5	NS	−0.07	1.3e−5	NS
LV global radial strain (%)	0.1	6.6e−12	NS	−0.07	8.6e−5	NS
LV global longitudinal strain (%)	−0.06	2.6e−5	NS	0.04	1.5e−2	NS
LV global circumferential strain (%)	−0.08	1.8e−9	NS	0.08	4.4e−6	NS
Myocardial mass (g)	−0.14	<2.2e−16	NS	0.16	<2.2e−16	NS
Myocardial wall thickness (mm)	−0.08	3.6e−9	NS	0.18	<2.2e−16	3.0e−2
CCI (SBP/LVESVi)	0.06	1.6e−5	NS	0.01	NS	NS
TNNI3 (troponin I) (NPX)	−0.02	4.4e−7	NS	0.09	<2.2e−16	<2.2e−16
NT-proBNP (NPX)	−0.08	<2.2e−16	8.9e−5	0.04	6.3e−15	6.3e−5
SBP (mmHg)	0.06	<2.2e−16	NS	0.07	<2.2e−16	3.0e−3
Urine albumin (mg/L)	0.17	<2.2e−16	1e−10	0.11	<2.2e−16	4.9e−13
HbA1c (mmol/mol)	−0.01	NS	NS	0.08	<2.2e−16	<2.2e−16

UKB analysis represents data from cohort of up to 52 705 participants. Adjusted model includes age, sex, creatinine, body mass index, cholesterol, systolic blood pressure (except when defining correlation with systolic blood pressure), prior MI, and prior hypertension.

CCI, cardiac contractility index; LV, left ventricle; LVEF, left ventricular ejection fraction; LVESVI, left ventricular end-systolic volume index; NPX, normalized protein expression units; SBP, systolic blood pressure.

We finally explored associations with clinically used circulating biomarkers of cardiometabolic status (*Table 4*). Lower ERBB3 was associated with higher concentrations of NT-proBNP (a marker of increased ventricular wall stress) and troponin I (a marker of cardiomyocyte injury), higher systolic blood pressure, and higher urinary microalbumin (a marker of diabetic microvascular disease), but did not correlate with HbA1c (a marker of glycaemic control); the association with NT-proBNP and urinary microalbumin persisted in adjusted analyses. Higher HSPA2 was also associated with higher NT-proBNP, troponin I, systolic blood pressure, urinary microalbumin, and HbA1c, all of which persisted in adjusted analyses. Overall these data suggest that ERBB3 and HSPA2 are associated with important elements of cardiometabolic status, in addition to their association with cardiovascular outcomes.

## 4. Discussion

We set out to identify myocardial transcriptomic signatures associated with DM and then validate hits in their plasma protein form to identify potential biomarkers. The transcriptomic hits we identified in RA and LV myocardium did not overlap, suggesting distinct pathological processes in these chambers, which may have important therapeutic implications and also emphasizes that RA biopsies are not a reliable proxy for studying LV disease. Many of the DEGs and GO terms we identified have not previously been linked to diabetic heart disease and will be important to explore in future studies. In this analysis, we focused on ERBB3, NRXN3, and HSPA2 as the only myocardial DEGs (all detected in LV) to show directionally concordant differential expression as a plasma protein and association with cardiovascular outcomes. In the whole UKB plasma proteomics cohort, including participants without diabetes, we found lower plasma ERBB3 and higher HSPA2 to be associated with impaired LV contractility, incident HF, and cardiovascular mortality. This is particularly interesting as cardiotoxicity is known to arise from cancer therapies targeting ERBB3 (and related family members) and because other studies have implicated HSPA2 in other forms of myocardial disease.^22–24^

While many animal studies have set out to explore how experimentally induced DM impacts upon cardiac gene expression, few human studies have addressed this issue, largely because of the challenge of accessing tissue, especially from the LV.^25^ The largest published analysis of DM-associated LV gene expression profiles was conducted by Liu *et al*., who compared seven people with type 2 DM and HF against 12 controls with only HF.^10^ They identified focal adhesions, vascular endothelial growth factor signalling, and mitogen-activated protein kinase signalling in pathway analyses. Our analysis did not highlight these pathways, possibly as Liu *et al*. focussed on DM associated with HF. The larger sample size of our transcriptomic cohort and validation of hits using an alternate cohort and technology suggest our main findings are likely to be robust. The wider transcriptomic themes that we identified could not be studied using UKB plasma proteomic data and so require external validation using emerging transcriptomic cohorts or alternate approaches. In particular, a focus on immunoglobulin gene expression (presumably in B lymphocytes and plasma cells) is warranted, given enriched GO terms in RA and LV, along with emerging roles of B lymphocytes in heart.^26,27^ Moreover, our data suggest that separate studies of atrial and ventricular myocardium are essential, given that the transcriptomic signature of DM differed between these sites. Our data also suggest that human post-mortem samples offer a valid route to biomarker and mechanism discovery, if technical factors are appropriately considered during analysis, potentially facilitating larger transcriptomic studies of LV myocardium which is difficult to acquire surgically.

Our finding that low circulating ERBB3 protein is associated with LV dysfunction and heart failure is further supported by data from patients receiving cancer therapeutics targeting ERBB2. Breast cancers commonly overexpress ERBB2 and agents that hinder epidermal growth factor signalling involving ERBB2 (e.g. trastuzumab) can improve survival.^28^ ERBB2 signals by heterodimerizing with other ERBB family members, including ERBB3^29^; indeed, the cancer therapeutic pertuzumab hinders ERBB2-ERBB3 heterodimerization.^30^ Trastuzumab and pertuzumab increase the risk of reduced LVEF or even HF;^31^ this risk is greater in people with DM.^32^ This led to the discovery that myocardial ERBB family receptors, including ERBB3, bind the ligand Neuregulin-1 (NRG1) that is released by cardiac endothelial cells in response to diverse stressors.^33^ Unfortunately UKB proteomic data do not include NRG1. However, recombinant forms of NRG1 have shown promise in augmenting LV contractility in HF, with some undergoing early phase clinical trials;^33,34^ they have also shown promise in a rat diabetic cardiomyopathy model.^35^ A recent multiomics study of human HF also implicated myocardial Erbb2 signaling;^36^ our analysis extends this to people without HF, provides broader coverage of the ERBB receptor family, and includes outcome data. The biological activity of circulating ERBB3 (e.g. as a decoy) is also unclear. *In vitro* studies suggest cleavage of ERBB3 by the protease ADAM17,^37^ but the role of ERBB family cleavage in myocardial biology is unknown. However, myocardial ADAM17 knockout mice develop less myocardial dysfunction associated with diet-induced diabetes, and it would be interesting to explore the role of ERBB3 in this phenotype.^38^ Irrespective of this uncertainty, we show that circulating ERBB3 can define people with early myocardial dysfunction and at risk of progression to HF. However, our data suggest that ERBB3 at best adds limited prognostic value to the established clinical biomarker NT-proBNP in all-comers. Whether it may offer greater value in defining patients at risk of cardiotoxicity from HER2-targetted cancer therapy and in identifying potential responders to recombinant NRG1 in clinical trials requires further research.

Little is known about the role of HSPA2 in myocardial biology. However, multiple studies have shown it to be increased in the myocardium at RNA and protein level in ischaemic, dilated, and hypertrophic cardiomyopathy vs. controls.^22–24^ It is a non-stress inducible member of HSP70 family, which support normal protein folding; it is released in extracellular vesicles during stressful conditions and may facilitate cell-cell communication.^39^ We show for the first time that it is increased in the myocardium of people with diabetes and that it may be a biomarker for CV death, although this needs more thorough assessment of any incremental value beyond NT-proBNP or value in targeting specific interventions.

Our study includes the largest described analysis of myocardial transcriptomic signatures associated with DM, followed by validation of hits in a very large plasma proteomic cohort that also allowed consideration of myocardial imaging and clinical outcomes for our principal hit. However, some limitations should be acknowledged. First, UKB proteomic data use Olink technology that quantifies proteins relative to the cohort mean concentration, rather than expressing these as absolute concentrations. This means that established cardiac biomarkers (troponin I and NT-proBNP) and novel plasma biomarkers have not been considered in light of clinically actionable thresholds, so further translational research is needed. It is also notable that this technology does not measure all circulating proteins, so it is not truly unbiased and contributed to our UKB analysis excluding 52 of 64 transcriptomic hits, which will need alternate validation approaches in future research. Second, our GTEx and UKB analyses could not stratify by type of DM due to the limitations of participant phenotyping (and also presumably statistical power). Therefore, while our findings apply to DM as a whole, they are probably heavily biased towards insights regarding type 2 DM. Since we explored UKB data in all comers (i.e. with and without diabetes), we expect that our findings will have broad relevance, but again, we cannot make extensive subgroup analyses due to a lack of statistical power. Similarly, our assessment of HF cannot discern the subtypes with reduced or preserved EF, and so future studies will need to consider these important groups. Third, plasma ERBB3 and HSPA2 do not necessarily reflect myocardial gene expression patterns, even though we have noted directionally concordant differences in people with DM and no evidence of differential expression in multiple other tissues. Future works using paired myocardial gene expression and plasma protein quantification are needed to define the relationship between these parameters. Finally, our study is observational and so cannot directly infer a causal role of ERBB3 or HSPA2 in myocardial dysfunction and adverse cardiovascular outcomes. Causal inference methods, such as Mendelian randomization, could be used to extend our analyses. However, we argue that more compelling data for the ERBB receptor family directly contributing to myocardial (dys)function come from the established cardiotoxicity of cancer therapies targeting these receptors and their signalling.

To conclude, we show that DM is associated with diverse myocardial transcriptomic signatures, which are broadly distinct in the RA and LV, although altered IgG immunoglobulin complex expression was noted in both sites. By assessing myocardial transcriptomic hits in their circulating protein form, we validated lower ERBB3 and higher HSPA2 in people with diabetes and found these to be associated with LV dysfunction, incident HF, and cardiovascular mortality. Together with wider evidence we discuss, these data suggest that ERBB signalling and HSPA2 may be important in the development of HF in people with diabetes, and warrant further exploration as biomarkers to target preventative therapies.

##  

**Translational perspective**

This work found diabetes is characterized by lower Erbb3 and higher Hspa2 mRNA expression in myocardium, with directionally concordant differences in their plasma protein concentration. These were linked to reduced LV contractility and future HF risk. Cancer therapies targeting ERBB3 are known to be cardiotoxic, while its ligand NRG1 may improve LVventricular contractility in HFrEF. High myocardial HSPA2 has also been found in diverse cardiomyopathies. Plasma ERBB3 and HSPA2 may help identify people at increased risk of developing HF and highlight unknown pathophysiological processes in diabetic heart disease.

## Supplementary Material

cvae181_Supplementary_Data

## Data Availability

The data underlying this article were accessed from the GTEx consortium (https://gtexportal.org) and UK Biobank (https://www.ukbiobank.ac.uk/) and are available to other scientists after application to these organisations.
